# Screening for Gestational Diabetes Mellitus in Early Pregnancy: What Is the Evidence?

**DOI:** 10.3390/jcm10061257

**Published:** 2021-03-18

**Authors:** Lore Raets, Kaat Beunen, Katrien Benhalima

**Affiliations:** 1 Department of Clinical and Experimental Endocrinology, KU Leuven, Herestraat 49, 3000 Leuven, Belgium; lore.raets@kuleuven.be (L.R.); kaat.beunen@kuleuven.be (K.B.); 2 Department of Endocrinology, University Hospital Gasthuisberg, KU Leuven, Herestraat 49, 3000 Leuven, Belgium

**Keywords:** pregnancy, gestational diabetes mellitus, early screening, diabetes

## Abstract

The incidence of gestational diabetes mellitus (GDM) is increasing worldwide. This has a significant effect on the health of the mother and offspring. There is no doubt that screening for GDM between 24 and 28 weeks is important to reduce the risk of adverse pregnancy outcomes. However, there is no consensus about diagnosis and treatment of GDM in early pregnancy. In this narrative review on the current evidence on screening for GDM in early pregnancy, we included 37 cohort studies and eight randomized controlled trials (RCTs). Observational studies have shown that a high proportion (15–70%) of women with GDM can be detected early in pregnancy depending on the setting, criteria used and screening strategy. Data from observational studies on the potential benefit of screening and treatment of GDM in early pregnancy show conflicting results. In addition, there is substantial heterogeneity in age and BMI across the different study populations. Smaller RCTs could not show benefit but several large RCTs are ongoing. RCTs are also necessary to determine the appropriate cut-off for HbA1c in pregnancy as there is limited evidence showing that an HbA1c ≥6.5% has a low sensitivity to detect overt diabetes in early pregnancy.

## 1. Introduction

Worldwide, the incidence of gestational diabetes mellitus (GDM) is increasing. This has a significant effect on the health of the mother and offspring. GDM is defined as diabetes diagnosed in the second or third trimester of pregnancy provided that overt diabetes early in pregnancy has been excluded [1]. There is no doubt that screening for GDM between 24 and 28 weeks is important to reduce the risk for adverse pregnancy outcomes such as large-for-gestational age infants (LGA) and preeclampsia [2,3]. There is a large variation in recommendations concerning screening for GDM in early pregnancy. The “American Diabetes Association” (ADA) recommends screening for overt diabetes at first prenatal visit, especially in women with risk factors. However, the ADA does not provide any specific recommendations concerning screening for GDM in early pregnancy [4]. “The International Association of the Diabetes and Pregnancy Study Groups” (IADPSG) initially recommended classification of GDM in early pregnancy when a fasting plasma glucose (FPG) ≥ 5.1 mmol/L occurs. However, the IADPSG criteria have not been validated for use in early pregnancy. Other associations such as the “International Federation of Gynecology and Obstetrics” (FIGO) recommend to screen universally in early pregnancy for diabetes and GDM [5]. In contrast, the “National Institute for Health and Care Excellence” recommends screening for early GDM if there are risk factors present, such as obesity, previous history of GDM, family history of diabetes (first-degree relative), previous macrocosmic baby or an ethnicity with a high prevalence of diabetes [6]. Early testing for overt diabetes will lead to the identification of hyperglycemia under the threshold of overt diabetes. These women could be labeled as early GDM based on IADPSG criteria, but there is a lack of evidence from randomized controlled trials (RCTs) on the potential benefits and harms of diagnosing and treating GDM in early pregnancy compared to treatment later in pregnancy. The ongoing controversy reflects in a lack of international consensus on screening for GDM in early pregnancy. The aim of this narrative review was therefore to evaluate the current evidence on screening and treatment for GDM in early pregnancy. In addition, we also reviewed pragmatic approaches to screening for glucose intolerance in pregnancy in a pandemic setting.

## 2. Materials and Methods

### 2.1. Data Sources and Search Strategies

Between November 2020 and December 2020, a literature search was conducted on PubMed. Cross-sectional studies, case–control studies, cohort studies, and RCTs were considered for this review. This is a narrative review. We did not perform a systematic review due to heterogeneity of studies and could therefore not perform a meta-analysis.

We used the following inclusion criteria:

1. The study population were pregnant women with early-onset GDM.
2. The control group could either be mothers with early GDM who were not treated or mothers with GDM diagnosed at 24–28 weeks of pregnancy (late-onset GDM).
3. The following comparisons were made: women with early GDM were compared to women with GDM diagnosed at 24–28 weeks or women with early GDM who received treatment before 24 weeks were compared to women with early GDM who did not receive treatment before 24 weeks.
4. The pregnancy outcomes studied were the development of GDM at 24–28 weeks, gestational weight gain, cesarean section, shoulder dystocia, preeclampsia, need for insulin treatment, LGA, neonatal intensive care unit (NICU) admission, neonatal hypoglycemia, preterm delivery and gestation age at delivery.

We excluded animal studies, descriptive designs (case series and case reports), studies with a low quality (no method section, no *p*-values mentioned), and articles written in a language other than English, French or Dutch. We did not limit our search to a specific population or ethnicity, or to a specific age category.

We used the following search strategies:

(“diabetes, gestational”(MeSH Terms) OR “Gestational diabetes”(Title/Abstract) OR “diabetes mellitus”(MeSH Terms) OR “diabetes mellitus” (Title/Abstract)) AND (“Blood Glucose” (MeSH Terms) OR “Blood Glucose” (Title/Abstract) OR “Insulin Resistance” (MeSH Terms) OR “hyperglycemia/blood” (MeSH Terms) OR “hyperglycemia” (Title/Abstract) OR “Glucose Intolerance” (MeSH Terms) OR “Glucose Tolerance Test” (MeSH Terms) OR “Glucose Tolerance Test” (Title/Abstract) OR “early screening” (Title/Abstract)) AND (“pregnancy trimester, first” (MeSH Terms) OR “First trimester pregnancy”(Title/Abstract))

In addition, we searched the reference lists of the selected articles and relevant reviews by hand. We also did a manual search for articles on screening for GDM during the COVID-19 Pandemic. We focused on articles that proposed a pragmatic approach on screening for diabetes and GDM in early pregnancy during the COVID-19 pandemic and compared the rate of missed cases of GDM between different screening tests and strategies.

### 2.2. Data Synthesis and Analysis

The extracted data included the study design, country, number of study participants, the diagnostic criteria used for GDM, adjustments that were made and pregnancy outcomes. We reported our results in a descriptive manner. A *p*-value <0.05 was considered significant.

## 3. Results

### 3.1. Search Results

**Figure 1:**
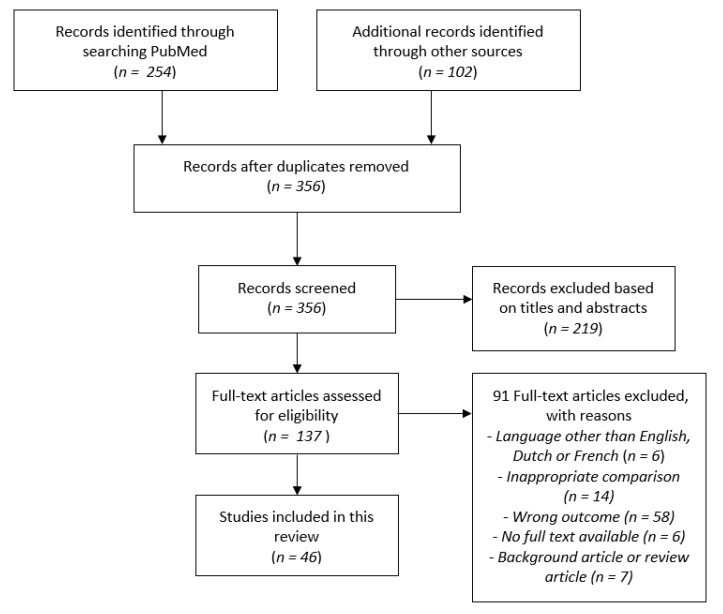
The literature search and selection process.

We identified 356 articles of which 137 articles were selected as possibly relevant. After examination of the full text, 46 studies were included in the current review (Figure 1).

### 3.2. Study Characteristics

The study characteristics are shown in Table 1, Table 2 and Table 3. In total, there were seven prospective cohort studies (15.6%), 29 retrospective cohort studies (63.0%), eight RCTs (17.8%), one post-hoc analysis (2.2%) and one population-based cohort study (2.2%). Six studies were performed in Asia (13.3%), 18 in Europe (39.1%), seven in America (15.6%), three in the Middle East (6.7%), four in Australia (8.9%), two in New Zealand (4.5%) and six multi-national studies (13.3%). All but one study was performed from 2000 onwards. Forty-one studies (89.9%) were performed from 2010 onwards. Twelve studies used the IADPSG criteria (26.7%). In total, 16 studies (34.9%) performed selective screening based on risk factors, while 30 studies (77.7%) used universal screening. Appendix A gives an overview of the diagnostic criteria used in the different studies.

**Table 1:** Observational studies.

Author, Year/Country (Ref.)	Design	Subjects (*N*)	Study Population	Timeframe Testing (Weeks)	GDM Criteria	Comparison	Main Results
De Muylder, 1984/Belgium [7]	Prospective cohort study	139	Hi risk	<24 weeks	3 h OGTT/O’Sullivan criteria	GDM diagnosis <24 weeks vs. GDM diagnosis 24–32 weeks vs. GDM diagnosis >32 weeks	Early GDM treated group had less complications such as preterm labor, preeclampsia and cesarean section
Bartha, 2000/Spain [8]	Prospective cohort study	3986	All pregnant women	First antenatal visit	50 g GCT and 3 h 100 g OGTT	Early-onset (most during 1st trimester) vs. late-onset GDM	Early GDM diagnosis represented a high-risk subgroup
Barahona, 2005/Spain [9]	Retrospective study	1708 offspring	Women with GDM	<24 weeks	50 g GCT and 3 h OGTT/2nd and 3rd Workshop Conference Criteria on GDM	GDM diagnosis <24 weeks vs. 24–30 weeks vs. >31 weeks	Early GDM diagnosis was a predictor of adverse maternal and neonatal outcome
Hawkins, 2008/US [10]	Retrospective cohort study	3334	All pregnant women	<24 weeks (Hi risk)	50 g GCT and 3 h 100 g OGTT/NDDG criteria	Diet-treated GDM <24 weeks (early diagnosis in hi risk population) vs. ≥24 weeks (routine diagnosis)	Twofold increased risk of preeclampsia in women with early diagnosis of diet treated GDM
Riskin, 2009/Israel [11]	Retrospective study	6129	Singleton pregnancies >24 weeks in mothers without ODIP or 1st FTFPG ≥5.8 mmol/L	<13 weeks	FTFPG/C&C criteria	FPG categories (<4.2 mmol/L, 4.2–4.4 mmol/L, 4.5–4.7 mmol/L, 4.8–5.0 mmol/L, 5.1–5.2 mmol/L, 5.3–5.5 mmol/L and 5.6–5.8 mmol/L)	Higher FTFPG in early pregnancy increased the risk of adverse pregnancy outcomes
Plasencia, 2011/Spain [12]	Retrospective study	1716	Singleton pregnancies	6–14 weeks	50 g GCT and 3 h 100 g OGTT/C&C criteria	GDM vs. non-GDM and GCT and OGTT results at 6–14 vs. 20–30 weeks	Effective diagnosis of GDM in the first trimester could be achieved by lowering the GCT and OGTT plasma glucose cut-offs
Corrado, 2012/Italy [13]	Retrospective study	738	Singleton pregnancies	<13 weeks	FTFPG/IADPSG criteria	FTFPG vs. 2 h 75 g OGTT early in the third trimester	FPG ≥5.1 mmol/L may be considered a highly predictive risk factor for GDM
Zhu W.W., 2013/China [14]	Retrospective cohort study	14,039	All pregnant women without ODIP	First antenatal visit (<24 weeks)	FPG/China GDM diagnosis criteria	6 FPG groups (<4.1, 4.1–4.59, 4.60–5.09, 5.10–5.59, 5.6–6.09, 6.10–6.99 mmol/L)	Only 30.3% of women who had an FPG of ≥5.1 mmol/L still had an FPG of ≥5.1 mmol/L at 24–28 weeks
Alunni, 2015/US [15]	Retrospective cohort study	2652	Singleton pregnancies in women without ODIP	≤24 weeks	HbA1c and FPG/HbA1c ≥5.7% or FPG ≥5.1 mmol/L at ≤24 weeks or C&C criteria	Early screening vs. standard two-step ACOG approach (1 h 50 g GCT followed by a 3 h 100 g OGTT/C&C Criteria)	Implementing early screening for GDM gave no significant difference in neonatal outcomes
Amylidi, 2015/ Switzerland [16]	Retrospective cohort study	208	Hi risk	<13 weeks	HbA1c/ADA and HAPO study guidelines	GDM vs. non-GDM (diagnosis based on one-step standardized 2 h 75 g OGTT between 24 and 28 weeks) and HbA1c ≥6% vs. <6%	Values HbA1c ≥6.0% in early pregnancy were predictive of GDM
Harreiter, 2016/ International [17]	Retrospective study	1035	Pregnant women with BMI ≥ 29.0 kg/m^2^	Early pregnancy	2 h 75 g OGTT/ WHO 2013 criteria	NGT vs. early GDM vs. DIP	Pre-pregnancy BMI was a significant predictor of early GDM and the only predictor among nulliparous women
Mañé, 2016/ Spain [18]	Prospective multi-ethnic cohort study	1228	Singleton pregnancies in women without ODIP	<13 weeks	HbA1c/≥5.9%	HbA1c ≥5.9% vs. HbA1c <5.9%	Early HbA1c ≥5.9% measurement identified women at high risk for poorer pregnancy outcomes
Osmundson, 2016/US [19]	Retrospective cohort study	2812	Singleton pregnancy >20 weeks	≤13^6/7^ weeks	HbA1c (prediabetes: 5.7–6.4%)	Prediabetic women (HbA1c of 5.7–6.4%) vs. women with a normal first trimester HbA1c (< 5.7%)	HbA1c early in pregnancy was a poor test to identify women who will develop GDM
Sweeting, 2016/Australia [20]	Retrospective cohort study	4873	Hi risk	<24 weeks	2 h 75 g OGTT/ADIPS diagnostic criteria	T2DM vs. GDM <12 weeks vs. GDM 12–23 weeks vs. GDM ≥24 weeks	Early GDM in high-risk women remains associated with poorer pregnancy outcomes
Sweeting, 2017/Australia [21]	Retrospective cohort study	3098	Hi risk	<24 weeks	HbA1c measurement at time of GDM diagnosis	Early GDM (<24 weeks) vs. standard GDM (≥24 weeks)	HbA1c >5.9% early in pregnancy identified an increased risk of LGA, macrosomia, C-section, and hypertensive disorders in standard GDM
Hosseini, 2018/Iran [22]	Prospective population-based cohort study	929	Singleton pregnancies	6–14 weeks	FPG/IADPSG	Normal pregnancy vs. early-onset GDM (6–14 weeks) vs. late-onset GDM (24–28 weeks)	Early-onset GDM was associated with poorer pregnancy outcomes
Ryan, 2018/UK [23]	Retrospective clinical audit of prospectively maintained database	576	Hi risk singleton pregnancies	11–13 weeks	FPG/SIGN 2010 thresholds	Routine vs. early screening	Early screening improved the pregnancy outcomes, such as emergency cesarean section, neonatal hypoglycemia and macrosomia.
Salman, 2018/Israel [24]	Retrospective cohort study	5030	Singleton pregnancies of women without ODIP	<13 weeks	FTFPG cut-off 5.3 mmol/L	Women with FTFPG < 5.3 mmol/L and FTFPG ≥ 5.3 mmol/L	FTFPG ≥5.3 mmol/L was an independent risk factor for adverse perinatal outcome
Bianchi, 2019/Italy [25]	Retrospective study	290	Hi risk	16–18 weeks	2 h 75 g OGTT (and FPG)/IADPSG criteria	Early (16–18 weeks) vs. standard (24–28 weeks) screening	Similar short-term maternal-fetal outcomes in both groups
Del Val López, 2019/Spain [26]	Retrospective study	1425	All pregnant women without ODIP	<13 weeks	FTFPG/O’Sullivan criteria	FTFPG <5.1 and ≥5.1 mmol/L (FTFPG vs. classical 2-step OGTT)	FTFPG was not a good substitute for conventional diagnosis of GDM in the second trimester
Mañé, 2019/Spain [27]	Retrospective analysis of a prospective observational cohort study	1228	Women with singleton pregnancy without ODIP	<13 weeks	FPG and HbA1c/Criteria unknown	FPG vs. HbA1c cut-off values	FTFPG levels were not a better predictor of pregnancy complications than HbA1c
Benhalima, 2020/Belgium [28]	Multi-centric prospective cohort study	2006	All pregnant women	6–14 weeks	FPG/IADPSG criteria	FPG ≥5.1–5.5 mmol/L in early pregnancy vs. FPG <5.1 mmol/L in early pregnancy	Group with increased FPG in early pregnancy had significantly more NICU admissions
Boriboonhirunsarn/Thailand, 2020 [29]	Retrospective cohort study	1200	All pregnant women	<24 weeks	50 g GCT and 100 g OGTT/ADA and ACOG recommendation	No GDM vs. early-onset GDM vs. late-onset GDM	Significant lower gestational weight gain and higher rates of preeclampsia, LGA infants, and NICU admission despite treatment for early-onset GDM
Clarke, 2020/Australia [30]	Retrospective cohort study	769	Hi risk with singleton pregnancy and without ODIP	<24 weeks	75 g 2 h OGTT/IADPSG criteria, as per the ADIPS guidelines	Early GDM (hi risk women diagnosed <24 weeks) vs. late GDM (women diagnosed ≥24 weeks)	Early pregnancy GDM was not associated with an adverse outcome
Cosson, 2020/France [31]	Retrospective study	523	Women with singleton pregnancy and without ODIP	<22 weeks	FPG/IADPSG criteria	Immediate care vs. no immediate care for early fasting hyperglycemia	Treating women with early fasting hyperglycemia, especially when FPG is ≥5.5 mmol/L, may improve pregnancy outcomes
Immanuel, 2020/International [32]	Post-hoc analysis of DALI study	869	Women with BMI ≥29 kg/m^2^ with singleton pregnancy and without ODIP	<20 weeks	HbA1c and 2 h 75 g OGTT/IADPSG criteria	HbA1c ≥5.7% vs. <5.7% group (prediabetes threshold)	Limited use of early pregnancy HbA1c for predicting GDM or adverse outcomes in overweight/obese European women
Jokelainen, 2020/Finland [33]	Population-based cohort study	1401	All singleton pregnancies without ODIP	12–16 weeks	2 h 75 g OGTT/FCCG	Early- vs. late-GDM vs. no GDM	Of the women who had early GDM based on the IADPSG/WHO criteria, 39.1% received the diagnosis of late GDM at the second OGTT
Liu, 2020/China [34]	Prospective cohort study	522	Singleton pregnancies	18–20 weeks	2 h 75 g OGTT/IADPSG-2015 guidelines	4 groups: NGT (no GDM diagnosis), EGDM (GDM diagnosis in only early OGTT), LGDM (GDM diagnosis in only standard OGTT) and GDM (GDM diagnosis in both OGTTs)	Early GDM diagnosis at 18–20 weeks is associated with adverse outcomes
Nakanishi, 2020/Japan [35]	Multicenter prospective cohort study	146	Hi risk without ODIP	<20 weeks	2 h 75 g OGTT/IADPSG criteria 2010	False-positive early GDM (early+/late-) vs. true GDM (early+/late+) (late = standard)	Of the 146 women diagnosed with early-onset GDM, 69 (47%) had normal 75 g OGTT values at 24–28 weeks of gestation.
Sesmilo, 2020/Spain [36]	Retrospective cohort study	6845	Singleton pregnancies in women without ODIP and available data	<13 weeks	FPG/NDDG criteria	FPG: ≤4.3, 4.4–4.6, 4.7–4.8 and ≥4.9 mmol/L	FTFPG is an early marker of GDM and LGA.

GDM: gestational diabetes mellitus; OGTT: oral glucose tolerance test; hi risk: high risk; GCT: glucose challenge test; NDDG: National Diabetes Data Group; ODIP: overt diabetes in pregnancy; FTFPG: first trimester fasting plasma glucose; HAPO: Hyperglycemia and Adverse Pregnancy Outcomes; C&C: Carpenter and Coustan; IADPSG: International Association of the Diabetes and Pregnancy Study Groups; FPG: fasting plasma glucose; HbA1c: hemoglobin A1C; ACOG: American Congress of Obstetricians and Gynecologists; ADA: American Diabetes Association; BMI: body mass index; WHO: World Health Organization; NGT: normal glucose tolerance; DIP: diabetes in pregnancy; ADIPS: Australian diabetes in pregnancy society; LGA: large-for-gestational age; C-section: cesarian section; SIGN: Scottish Intercollegiate Guidelines Network; NICU: neonatal intensive care unit; T2DM: type 2 diabetes mellitus; FCCG: Finnish Current Care Guideline; EGDM: early-onset gestational diabetes; LGDM: late-onset gestational diabetes; DALI: Diabetes and Pregnancy Vitamin D And Lifestyle Intervention for Gestational Diabetes Mellitus Prevention.

**Table 2:** Randomized controlled trials.

Author, Year/Country (Ref.)	Subjects (*N*)	Study Population	Timeframe Testing (Weeks)	GDM Criteria	Comparison	Main Results
Osmundson, 2016/US [37]	83	Women with singleton pregnancy and without ODIP	<14.0 weeks	HbA1c/between 5.7 and 6.4%	Usual care vs. early treatment for GDM with diet, BG monitoring, and insulin as needed	Early treatment did not significantly reduce the risk of GDM except in non-obese women
Hughes, 2018 (PINTO feasibility study)/New Zealand [38]	47	Women with singleton pregnancy and without ODIP	<14.0 weeks	HbA1c/between ≥5.9 and 6.4%/2 h 75 h OGTT New Zealand criteria	Standard care vs. early intervention in pregnancies complicated by prediabetes	First results expected in 2021
Simmons, 2018 (ToBOGM pilot study)/Australia [39]	79	Hi risk women with singleton pregnancy	<20.0 weeks (4–19.6 weeks)	2 h 75 g OGTT/IADPSG criteria	Women with booking GDM receiving immediate (clinical referral or ongoing treatment) vs. deferred (no) treatment vs. women without booking GDM (“decoys”)	More NICU admission in the early GDM group with a tendency for more SGA but less LGA
Simmons, 2018 (ToBOGM study protocol)/International [40]	4000	Hi risk women with singleton pregnancy	<20.0 weeks (4–19.6 weeks)	2 h 75 g OGTT/2014 Australasian Diabetes-in-Pregnancy Society criteria for pregnant women with GA 24–28 weeks	Intervention (immediate treatment) vs. control (no treatment) vs. decoys (NGT but undergo all procedures) vs. non-active (NGT and records reviewed postnatally)	First results expected mid-2021
Vinter, 2018 (LiP study)/Denmark [41]	90	Obese pregnant women (BMI 30–45 kg/m^2^) with singleton pregnancy	12–15 weeks	2 h 75 g OGTT/IADPSG Criteria	Lifestyle intervention vs. SoC	Lifestyle intervention was not effective in improving obstetric or metabolic outcomes
Roeder, 2019 (RCT)/US [42]	157	Women with hyperglycemia and singleton pregnancy without ODIP	≤15.0 weeks	HbA1c and/or FPG, respectively, 5.7–6.4% and/or 5.1–6.9 mmol/L	Early pregnancy vs. 3rd trimester treatment of hyperglycemia	Treatment in early pregnancy did not improve maternal or neonatal outcomes significantly
Harper, 2020 (EGGO study)/US [43]	922	Obese women (BMI ≥30 kg/m^2^) without ODIP and history of bariatric surgery	14–20 weeks	2-step method: 1 h 50 g GCT followed by a 3 h 100 g OGTT/C&C criteria	Early GDM screening (14–20 weeks) vs. routine screening (24–28 weeks)	Early GDM screening in obese women did not reduce the composite perinatal outcomes, such as macrosomia, C-section and shoulder dystocia
Hung-Yuan Li (TESGO study)/Taiwan (NCT03523143)	2068	Singleton pregnancy without ODIP	18–20 weeks	75 g 2 h OGTT/IADPSG criteria	Early screening group (18–20 weeks) vs. standard screening group (24–28 weeks)	Results expected beginning of 2021

ODIP: overt diabetes in pregnancy; HbA1c: hemoglobin A1c; GDM: gestational diabetes mellitus; BG: blood glucose; PINTO: Pre diabetes in pregnancy, can early intervention improve outcomes; OGTT: oral glucose tolerance test; Hi risk: high risk; ToBOGM: Treatment of Booking Gestational diabetes Mellitus; IADPSG: International Association of the Diabetes and Pregnancy Study Groups; NICU: neonatal intensive care unit; SGA: small-for-gestational age; LGA: large-for-gestational age; GA: gestational age; NGT: normal glucose tolerance; LIP: Lifestyle in Pregnancy; BMI: body mass index; SoC: standard of care; FPG: fasting plasma glucose; GCT: glucose challenge test; C&C: Carpenter and Coustan; C-section: cesarean section; EGGO: Early Gestational Diabetes Screening in the Gravid Obese Woman; TESGO: The Effect of Early Screening and Intervention for Gestational Diabetes Mellitus on Pregnancy Outcomes.

### 3.3. Screening for Overt Diabetes in Early Pregnancy

The prevalence of type 2 diabetes (T2DM) in women of childbearing age is increasing. Since T2DM is often asymptomatic at the beginning and women with severe hyperglycemia early in pregnancy are at high risk for adverse pregnancy outcomes, timely detection and treatment of diabetes is needed. Most international associations such as the IADPSG, the ADA and the World Health Organization (WHO) recommend therefore to screen for overt diabetes at the first antenatal visit using an FPG, HbA1c or 75 g oral glucose tolerance test (OGTT) with the same cut-offs as for non-pregnant populations. The measurements of FPG and HbA1c should ideally be repeated twice to confirm the diagnosis of overt diabetes. HbA1c can be used to screen for diabetes but not to screen for GDM due to the very low sensitivity [44]. Measurement of fasting glycemia has a higher sensitivity than HbA1c to screen for diabetes. On the other hand, HbA1c has the advantage that it can be performed in the non-fasting state. An observational study from New Zealand [45] showed that an HbA1c ≥5.9% identified all women with diabetes who completed an OGTT before 20 weeks of pregnancy and this also identified a group at significantly increased risk for adverse pregnancy outcomes, such as preeclampsia and shoulder dystocia. In addition, the study demonstrated that an HbA1c ≥6.5% would have missed almost half of these women. These data suggest therefore that the currently recommended HbA1c is too high for screening purposes in pregnancy. However, large RCTs are required to confirm these results.

### 3.4. Screening for GDM in Early Pregnancy

As shown in Table 1, numerous observational studies were performed over the years. These studies show conflicting results. In general, most studies show that women with early GDM are at high risk for adverse pregnancy outcomes but treatment of GDM early in pregnancy compared to later in pregnancy does not always translate into improved outcomes. Seven studies reported an improved pregnancy outcome by treatment of early-onset GDM [7,9,10,29,30,31,34]. Barahona et al. [9] showed that diagnosing GDM early in pregnancy is a predictor of adverse maternal and neonatal outcomes, such as pregnancy-induced hypertension, insulin treatment during pregnancy, preterm birth, hyperbilirubinemia and perinatal mortality. More recently, Cosson et al. [31] reported that women who received initial care vs. those who did not, were more likely to be insulin-treated during pregnancy (58.0% vs. 20.9%, respectively; *p* < 0.00001), gained less gestational weight (8.6 ± 5.4 kg vs. 10.8 ± 6.1 kg, respectively; *p* < 0.00001), had a lower rate of preeclampsia (1.2% vs. 2.6%, respectively; adjusted odds ratio (aOR): 0.247 (0.082–0.759), *p* = 0.01), and similar rates of LGA infants and shoulder dystocia. A very recent study from Thailand showed that early GDM women had a high risk for adverse pregnancy outcomes with higher rates of preeclampsia, LGA infants, and NICU admission [29].

However, five studies described no beneficial effect of early diagnosing or treatment of GDM on maternal or neonatal outcomes [15,20,22,25,30]. Both Alluni et al. [15] and Bianchi et al. [25] showed that patients diagnosed and treated for early-onset GDM were more prone to be insulin-treated during pregnancy but showed no differences in neonatal outcomes such as small-for-gestational age (SGA) infants, cesarean sections, macrosomia, and LGA. An Australian study demonstrated also that early diagnosis and intervention had no effect on pregnancy outcomes [21]. This was confirmed by a recent Australian study showing no differences in pregnancy outcomes between early-onset GDM and late-onset GDM [30].

RCTs are needed to determine whether treating early-onset GDM improves pregnancy outcomes compared to standard treatment of GDM at 24–28 weeks of pregnancy. Table 2 gives an overview of the (ongoing) RCTs. The largest RCTs such as the “Prediabetes in pregnancy, can early intervention improve outcomes” PINTO study, the “Treatment of Booking Gestational diabetes Mellitus” (ToBOGM) study and the “Effect of Early Screening and Intervention for Gestational Diabetes Mellitus on Pregnancy Outcomes” (TESGO) study are still ongoing. Results are expected at the earliest in 2021. The ToBOGM study, the “Early Gestational Diabetes Screening in the Gravid Obese Woman” (EGGO) study and “Lifestyle in Pregnancy” (LiP) study focused on high-risk populations and obese women. In contrast, the TESGO and PINTO studies included also lower risk women.

A small RCT showed that early treatment of mild hyperglycemia (women with an HbA1c of 5.7–6.4%) did not reduce the risk of GDM, except for non-obese women [37]. The pilot study of ToBOGM [39] demonstrated that early treatment may have both benefits and harms for mother and offspring. NICU admission was highest in the treated early GDM group (36% vs. 0% *p* = 0.043), driven by a higher rate of SGA infants. Women who received no treatment for early-onset GDM had more LGA infants (0% vs. 33% *p* = 0.030). The LiP study [41] focused on the effect of lifestyle intervention vs. standard care for obese women with early GDM. They found that lifestyle intervention in early pregnancy did not improve obstetric or metabolic outcomes. In addition, the EGGO study [43] showed no effect on the composite perinatal outcomes in obese women who had early screening for GDM. Similarly, Roeder et al. [42] did not find any improvement in maternal and neonatal outcomes after treatment in early pregnancy.

### 3.5. Criteria to Define GDM in Early Pregnancy

Of all observational studies (Table 1), 15 studies discussed the diagnostic criteria for GDM early in pregnancy [11,12,13,14,16,18,21,24,26,27,28,31,32,35,36]. Riskin et al. showed that first trimester fasting glucose levels (FTFPG) in the non-diabetic range resulted in a higher risk for adverse pregnancy outcomes, such as more cesarian sections, LGA and macrosomia. These findings were confirmed by a recent Belgium study [28] demonstrating more NICU admissions in the high FTFPG group (FPG ≥ 5.1–5.5 mmol/L). A large Chinese study showed that an FTFPG of 6.1–7.0 mmol/L in early pregnancy is a strong predictor for GDM later in pregnancy [14]. In contrast, an FTFPG ≥ 5.1 mmol/L (GDM according to IADPSG criteria) was not a good predictor for GDM in their population. Several other studies (including studies in European populations) confirmed that ≥ 5.1 mmol/L is a poor predictor for GDM early in pregnancy [26,28]. A Belgium study [28] for instance demonstrated that only 37% of all women with an FTFPG ≥ 5.1–5.5 mmol/L, developed GDM based on the OGTT later in pregnancy. A French study proposed to use an FTFPG ≥ 5.5 mmol/L to start treatment for GDM in early pregnancy, as they demonstrated improved pregnancy outcomes in their population [31].

Few studies evaluated HbA1c in early pregnancy to diagnose GDM. These studies showed that an HbA1c ≥ 5.9% identifies women at high risk for adverse pregnancy outcomes independently of GDM diagnosis later in pregnancy [21,27].

A small RCT showed that early treatment of women with a first trimester HbA1c of 5.7–6.4% did not significantly reduce the risk of GDM, except in non-obese women [37]. Roeder et al. [42] used HbA1c ≥ 5.7% and/or an FTFPG ≥ 5.1 mmol/L to identify women with hyperglycemia early in pregnancy. Treatment in early pregnancy did not improve maternal or neonatal outcomes. Only 19% of this cohort developed GDM later in pregnancy [42]. In contrast, the ToBOGM pilot trial reported that 89% of untreated women (with an FPG ≥ 5.1 mmol/L early in pregnancy) had GDM at 24–28 weeks [39].

### 3.6. Screening for Diabetes and GDM in Early Pregnancy in COVID Times

Due to the COVID-19 pandemic, screening for GDM using OGTT’s might lead to an increased risk for exposure to the virus. Six large observational studies describe how screening for GDM could be organized in a pragmatic way using blood tests, and risk calculators applied to underlying risk factors (Table 3).

**Table 3:** Screening for diabetes and GDM (early) in pregnancy in COVID times.

Article	Pragmatic Approach	Main Results
Thangaratinam, 2020 [49]	Test strategy: - Early GDM screening: additional tests at booking (HbA1c and RPG) to detect overt diabetes and identify those at highest risk for GDM Suggested thresholds and actions: - HbA1c ≥ 6.5% or RPG ≥ 11.1mmol/L: treat as preexisting diabetes. - HbA1c 5.9–6.5% or RPG 9–11 mmol/L: consider managing using the GDM pathway. - Avoid OGTT at 24–28 weeks and instead offer HbA1c along with FPG or RPG if fasting values are not availableSuggested thresholds and actions: Suggested thresholds and actions: HbA1c ≥ 5.7% or FPG ≥ 5.6 mmol/L or RPG ≥ 9 mmol/L: treat as GDM.	Using FPG alone will only pick up about half of all women with GDM, based on NICE or IADPSG criteria. Combining FPG with HbA1c may improve the detection rate. Maintaining existing FPG thresholds may be preferable, and services may consider lower thresholds consistent with the IADPSG diagnostic criteria (FPG ≥ 5.1), if resources allow.
Torlone, 2020 [50]	Screening for overt diabetes: - FPG ≥6.9 mmol/L - RPG ≥11.1 mmol/L - HbA1c ≥6.5% A single value can be considered valid during COVID-19 emergency Screening for GDM: risk factors assessment - Women at high risk for GDM: FPG ≥5.1 mmol/L at 16–18 weeks → GDM - Women at high risk for GDM: FPG ≤5.1 mmol/L at 16–18 weeks → FPG at 24–28 weeks ≥5.1 mmol/L → GDM - Women at medium risk for GDM: FPG ≥5.1 mmol/L at 24–28 weeks → GDM	A fasting glucose value can be considered diagnostic for GDM only when it is obtained at the gestational age when the OGTT should have been carried out, i.e., between 16 and 18 weeks in high-risk pregnant women or between 24 and 28 weeks in medium-risk women.
McIntyre, 2020 (Diagnosis and management GDM during COVID-19) [47]	Early in pregnancy: all guidelines: HbA1c ≥ 5.9% Standard screening (24–28 weeks): *UK:* at risk; GDM if HbA1c ≥ 5.7% and/or FVPG ≥ 5.6 mmol/L and/or Random VPG (not preferred) ≥ 9.0 mmol/L *CAN:* GDM if HbA1c ≥ 5.7% and/or Random VPG ≥ 11.1 mmol/L *AUS:* fasting VPG: Fasting VPG < 4.7 mmol/L = normal Fasting VPG 4.7–5.0 mmol/L = OGTT, WHO 2013 criteria Fasting VPG ≥ 5.1 mmol/L = GDM	Detecting only those with marked hyperglycemia
McIntyre, 2020 (Testing for GDM during COVID-19) [46]	*UK:* Risk factor based; no OGTT; GDM if HbA1c ≥ 5.7% and/or FVPG ≥ 5.6 mmol/L and/or Random VPG (not preferred) ≥ 9.0 mmol/L *CAN*: universal testing; no OGTT; GDM if HbA1c ≥ 5.7% and/or Random VPG ≥ 11.1 mmol/L *AUS*: fasting VPG: Fasting VPG < 4.7 mmol/L = normal Fasting VPG 4.7–5.0 mmol/L = OGTT, WHO 2013 criteria Fasting VPG ≥ 5.1 mmol/L = GDM	All post COVID-19 modified pathways reduced GDM frequency. Missed GDM’s in Canadian women gave similar rates of pregnancy outcomes. Using UK modifications, missed GDM group was at slightly lower risk. Using the Australian modifications, missed GDM group was at substantially lower risk.
Meek, 2020 [48]	To evaluate the diagnostic and prognostic performance of alternative diagnostic strategies to 2 h 75 g OGTTs: HbA1c, RPG and FPG GDM diagnosis: criteria of the UK National Institute for Health and Care Excellence and IADPSG criteria	RPG at 12 weeks, and FPG or HbA1c at 28 weeks identify women with hyperglycemia at risk of suboptimal pregnancy outcomes.
Seshiah, 2020 [51]	The “single test procedure” for diagnosing GDM: 2 h PG ≥ 7.8 mmol/L with 75 g oral glucose administered to a pregnant woman in the fasting or non-fasting state, without regard to the time of the last meal (glucose load can also be taken at home and the pregnant woman can visit the hospital 2 h after the glucose ingestion to give a single sample for plasma glucose estimation)	The economical and evidence based “single test procedure” of DIPSI is most appropriate for screening during the COVID pandemic as performing OGTTs is resource intensive, the fasting state is impractical with very high dropout rate.

GDM: gestational diabetes mellitus; HbA1c: hemoglobin A1c; RPG: random plasma glucose; OGTT: oral glucose tolerance test; FPG: fasting plasma glucose; NICE: National Institute for Health and Care Excellence; IADPSG: International Association of the Diabetes and Pregnancy Study Groups; VPG: venous plasma glucose; UK: United Kingdom; CAN: Canada; AUS: Australia; WHO: World Health Organization; PG: plasma glucose; DIPSI: Diabetes in Pregnancy Study Group India.

McIntyre et al. [46,47] described the diagnosis and management of GDM during COVID-19 in Australia, Canada and the United Kingdom (UK). For early screening, the guidelines were similar in the different countries, with an HbA1c ≥ 5.9% considered as hyperglycemia. For diagnosing GDM at 24–28 weeks, each country had a slightly different approach. The UK invited only women at high risk. GDM was diagnosed if HbA1c ≥ 5.7% and/or fasting venous plasma glucose (FVPG) ≥ 5.6 mmol/L and/or random venous plasma glucose (VPG) (not preferred) ≥ 9.0 mmol/L. Canada recommended universal screening. GDM was diagnosed if HbA1c ≥ 5.7% and/or random VPG ≥ 11.1 mmol/L. Australia used universal testing with an initial FVPG. If FVPG was between 4.7 and 5.0 mmol/L, an OGTT was performed. If FVPG ≥ 5.1 mmol/L, then GDM was diagnosed immediately.

Meek et al. [48] reported that random plasma glucose (RPG) at 12 weeks, and FPG or HbA1c at 28 weeks identifies women with hyperglycemia at risk of suboptimal pregnancy outcomes. When an OGTT is not possible, as an alternative RPG, FPG and HbA1c are recommended. Thangaratinam et al. [49] suggested additional tests at booking to detect overt diabetes and identify those at highest risk for GDM. HbA1c ≥ 6.5% or RPG ≥ 11.1 mmol/L is considered as pre-existing diabetes. As the recommended antenatal routine booking blood tests are often not performed in the fasting state, a pragmatic approach was suggested with the use of RPG. GDM can be diagnosed by HbA1c 5.9–6.4% or RPG 9–11 mmol/L to diagnose diabetes. The following thresholds for diagnosing GDM were suggested: HbA1c ≥ 5.7%, FPG ≥ 5.6 mmol/L or RPG ≥ 9 mmol/L.

To conclude, Italian recommendations [50] propose that if an OGTT cannot be safely performed, screening for GDM should be based on risk factors and the FPG value. Women with high risk factors should be tested at 16–18 weeks and an FPG ≥5.1 mmol/L is diagnosed as GDM. Women with high risk factors and an FPG ≤5.1 mmol/L or women with medium risk factors should be tested at 24–28 weeks. If FPG ≥5.1 mmol/L at 24–28 weeks, the woman will be diagnosed with GDM.

## 4. Discussion

### 4.1. Summary of Findings

In this narrative review, we demonstrate that there is need for clear guidelines and criteria concerning overt diabetes and early screening for GDM. The HbA1c threshold of diabetes as currently recommended is probably too high to detect all women with overt diabetes in early pregnancy. Furthermore, observational studies show conflicting results on the effects of screening and treatment of GDM in early pregnancy. It is also not clear which diagnostic criteria should be used to define GDM in early pregnancy. Evidence from large RCTs is needed to evaluate whether treatment has a beneficial effect on maternal and neonatal outcomes, without increased risk for harm (such as increased risk for SGA infants). Large RCTs such as the TOBOGM study will also help to inform which diagnostic criteria are appropriate to use for GDM in early pregnancy.

The COVID-19 pandemic has brought us additional challenges. OGTT’s could often not be performed as they involve high exposure risks and health service burden. Different guidelines have proposed pragmatic approached to screening with HbA1c, FPG or even RPG as an alternative during the pandemic.

### 4.2. Results in Relation to What We Already Know

Screening for overt diabetes in early pregnancy is necessary. At the moment, the threshold for diagnosing overt diabetes is an HbA1c ≥6.5%. However, there is limited data suggesting that in pregnancy the cut-off of HbA1c should be lowered to ≥5.9% to identify all women with diabetes as this identifies a population at high risk for adverse pregnancy outcomes. A threshold of ≥6.5% would have missed half of these women [45].

Screening and treatment of GDM between 24 and 28 weeks of pregnancy is widely accepted. This is beneficial to reduce adverse pregnancy outcomes. More women are identified with mild hyperglycemia in early pregnancy due to increased screening for overt diabetes. Observational studies show conflicting results as to whether screening and treatment of GDM in early pregnancy is beneficial compared to screening later in pregnancy. Some studies have shown that despite treatment, early-onset GDM women have more adverse pregnancy complications than late-onset GDM women, while other studies demonstrated similar short-term obstetrical outcomes in both groups and improved outcomes in the early screening group. At this moment, we can only speculate whether the fact that early treatment of GDM often only leads to similar pregnancy outcomes might represent success rather than failure. We cannot exclude that treatment of this group later in pregnancy might lead to more adverse pregnancy outcomes. In addition, studies had substantial heterogeneity in maternal age in pregnancy and BMI. In developed countries, increasing maternal age in pregnancy disposes to higher insulin resistance, whereas most pregnant women are younger in developing countries. Data are needed from well-designed RCTs. In the meantime, treatment of mild hyperglycemia in early pregnancy remains controversial due to lack of evidence from large RCTs supporting any benefit of treatment of GDM before 24 weeks of pregnancy. Moreover, a diagnosis of GDM could also be associated with increased medicalization of pregnancy (with more inductions and cesarean sections) and an increased risk for SGA infants due to overtreatment. Furthermore, it remains unclear which diagnostic criteria should be used to define GDM in early pregnancy, and whether universal or selective screening should be used to detect GDM before 24 weeks. Many studies evaluated high risk populations and there was also a high heterogeneity in the risk factors used across the different studies to screen for GDM in early pregnancy. Several studies have shown that FPG in early pregnancy is a poor predictor for GDM later in pregnancy. The IADPSG criteria might therefore not be appropriate to use in early pregnancy.

### 4.3. Practical Implications

As for maternal and neonatal outcomes, smaller RCTs did not show benefits of early screening and treatment of GDM. The EGGO and LIP studies were performed in obese populations and showed no improvement in pregnancy outcomes in the group who received treatment early in pregnancy compared to treatment later in pregnancy. These data suggest that future studies should focus on interventions starting pre-pregnancy in obese women. The pilot study of the TOBOGM study showed both benefits and harms of early treatment of GDM in a high-risk population. The treated group had a lower LGA rate but more NICU admissions, mainly due to a higher SGA rate. SGA can be a consequence of overtreatment [52], or insufficient gestational weight gain [53]. This highlights the need for data from large RCTs. The results of several large ongoing RCTs are expected for mid-2021 at the earliest. Many studies were conducted in a high-risk population. It is therefore also important to have evidence on the potential benefit or harm of screening for early GDM in low-risk populations and when using a universal screening strategy.

As we are waiting for stronger evidence from RCT’s, we do currently not recommend screening and treatment of GDM before 24–28 weeks of gestation in our center [54]. In line with the Flemish consensus of 2019 on screening for GDM [54], we recommend to universally screen for overt diabetes in early pregnancy. In addition, we propose a pragmatic approach for women who are diagnosed with mild hyperglycemia (FPG 5.5–6.9 mmol/L) in early pregnancy. We do not label these women as early GDM but advise follow-up with a dietician early in pregnancy (since these women are often overweight) and provide screening for GDM with an OGTT at 24 weeks of pregnancy [54].

The first results of the RCTs also show that treatment of women with HbA1c ≥ 5.7% and/or an FTFPG ≥ 5.1 mmol/L in early pregnancy, does not improve pregnancy outcomes [42]. HbA1c alone was not a good predictor for GDM early in pregnancy, because of the low sensitivity. It should always be used with other standard diagnostic tests for GDM, as was also demonstrated by the systematic review of Renz et al. [44]. FPG level generally further decreases by the end of the first trimester. Using FPG early in pregnancy, can lead to false positive results. Several studies have shown that an FPG level is a poor predictor for GDM with a sensitivity and specificity of 33–66% to predict GDM later in pregnancy. In contrast, the ToBOGM pilot study reported that 89% of the untreated women with early GDM in their study developed GDM at 24–28 weeks of gestation [39]. However, an OGTT was used in early pregnancy to screen for GDM (not only FTFPG) and this study evaluated a high-risk population.

Studies did not always report the pre-analytical method of collecting blood for FPG. Correct pre-analytic sampling of plasma glucose is important to prevent glycolysis and to prevent false negative results. A recent study of O’Malley showed that fluoride tubes must be stored on ice or must be centrifuged within 30 min to prevent glycolysis [55].

Since 2020, we have been faced with the impact of the COVID-19 pandemic on health care delivery. This might also impact screening for GDM and diabetes in pregnancy. There is a need to balance the sometimes-competing requirement of lowering the risk of direct viral transmission against the potential adverse impact of service changes. A pragmatic approach to screening for GDM is advised if an OGTT is not feasible. As an alternative, FPG, RPG and HbA1c, can be used. Women with a high-risk profile or with a history of GDM need to be closely monitored. It is important that usual guidelines and care will be re-evaluated as soon as possible [46,47,48,49,51].

### 4.4. Strengths and Limitations

A strength of this overview is that we performed an extensive narrative review including 45 studies evaluating the evidence on screening for GDM from both observational studies and RCTs. We provided an updated and detailed overview of the different observational and (ongoing) RCT’s, including data on timing of screening, the diagnostic criteria used for GDM, the screening strategy and comparator used. In addition, we highlighted the heterogeneity in risk factors used for selective screening in early pregnancy. However, our review also has several limitations. We did not perform a systematic review and could therefore not perform a meta-analysis. We could therefore also not assess the risk of bias of individual studies and did not contact the authors for obtaining missing and unpublished data. In addition, we did not assess the pre-analytical method of collecting blood for FPG determination.

## 5. Conclusions

Observational studies show conflicting results as to whether screening and treatment of GDM in early pregnancy is beneficial. However, most studies show that women with early GDM are at high risk of adverse pregnancy outcomes. A slight majority of relevant observational studies report an improved pregnancy outcome by treatment of early-onset GDM. However, so far, RCTs have not provided conclusive evidence of the beneficial effects of early treatment. Evidence from large RCTs is urgently needed, also evaluating lower risk populations to determine appropriate early-pregnancy OGTT thresholds for the diagnosis of GDM, and to assess the impact of early treatment on obstetrical outcomes and long-term offspring health. RCTs are also necessary to determine the appropriate cut-off of HbA1c in early pregnancy to identify women at risk for adverse pregnancy outcomes. Therefore, we currently recommend a pragmatic approach for women diagnosed with mild hyperglycemia in early pregnancy. A pragmatic approach to screen for GDM can be implemented during the COVID-19 pandemic, using FPG, RPG or HbA1c. However, routine guidelines and care must be re-evaluated as soon as possible.

## Data Availability

No new data were created or analyzed in this study. Data sharing is not applicable to this article.

## Appendix A

**Table A1:** This table gives an overview of the gestational diabetes mellitus diagnosis criteria used in this review.

Criteria	OGTT	FPG	1 h	2 h	3 h	Number of Abnormal Values
C&C	100 g	≥5.3 mmol/L	≥10 mmol/L	≥8.6 mmol/L	≥7.8 mmol/L	≥2
NDDG	100 g	≥5.8 mmol/L	≥10.5 mmol/L	≥9 mmol/L	≥8 mmol/L	≥2
IADPSG, WHO 2013	75 g	≥5.1 mmol/L	≥10 mmol/L	≥8.5 mmol/L		≥1
ADA	100 g	≥5.3 mmol/L	≥10 mmol/L	≥8.6 mmol/L	7.8 mmol/L	≥2
WHO 1999	75 g	≥6 mmol/L		≥7.8 mmol/L		≥1
Finnish Diabetes Association	75 g	>5.3 mmol/L	>10 mmol/L	>8.6 mmol/L		≥1
New-Zealand criteria	75 g	≥5.5 mmol/L		≥9 mmol/L		≥1
O’Sullivan criteria	100 g	≥5 mmol/L	≥9 mmol/L	≥7.8 mmol/L	≥6.9 mmol/L	≥2
Chinese GDM criteria	75 g	≥5.1 mmol/L	≥10 mmol/L	≥8.5 mmol/L		≥1
ADIPS	75 g	≥5.1 mmol/L	≥10 mmol/L	≥8.5 mmol/L		≥1
Scottish Intercollegiate Guidelines Network	75 g	≥5.1 mmol/L	≥10 mmol/L	≥8.5 mmol/L		≥1
DIPSI	75 g			≥7.8 mmol/L		≥1

OGTT: oral glucose tolerance test; FPG: fasting plasma glucose; C&C: Carpenter and Coustan; NDDG: National Diabetes Data Group; IADPSG: International Association of the Diabetes and Pregnancy Study Groups; WHO: World Health Organization; ADA: American Diabetes Association; ADIPS: Australian Diabetes in Pregnancy society; DIPSI: Diabetes in Pregnancy Study Group India.
